# Supplementary material for: Consolidated BRCA1/2 Variant Interpretation by MH BRCA Correlates with Predicted PARP Inhibitor Efficacy Association by MH Guide
Source: Int J Mol Sci. 2020 May 29;21(11):3895. doi: 10.3390/ijms21113895 (PMC7312854; doi:10.3390/ijms21113895)
Supplement: Supplementary file 1 [file ijms-21-03895-s001.zip › Figure S1-S3.pdf]

Supplemental Figure 1

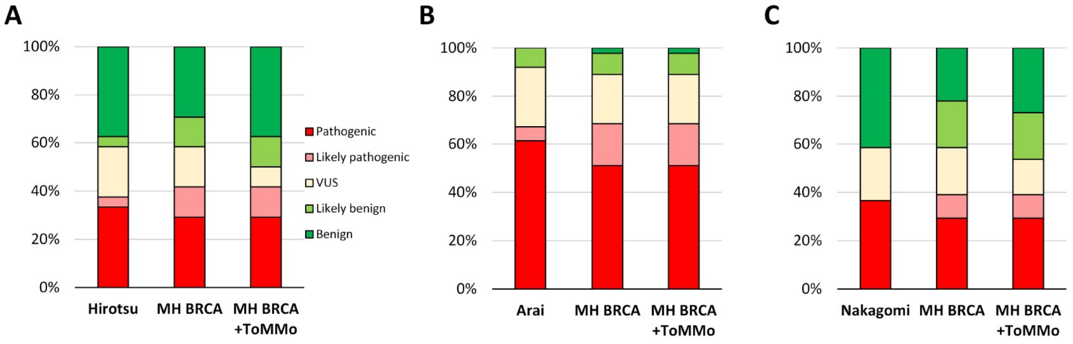

Figure S1. Comparison of MH BRCA to Japanese HBOC studies. Classifications of *BRCA1/2* variants were compared to results in MH BRCA with or without the Japanese genomic cohort data of ToMMo. Data were taken from the studies of (A) Hirotsu et al. [13] ($n = 24$), (B) Arai et al. [12] ($n = 137$), and (C) Nakagomi et al. [14] ($n = 41$).

Supplemental Figure 2

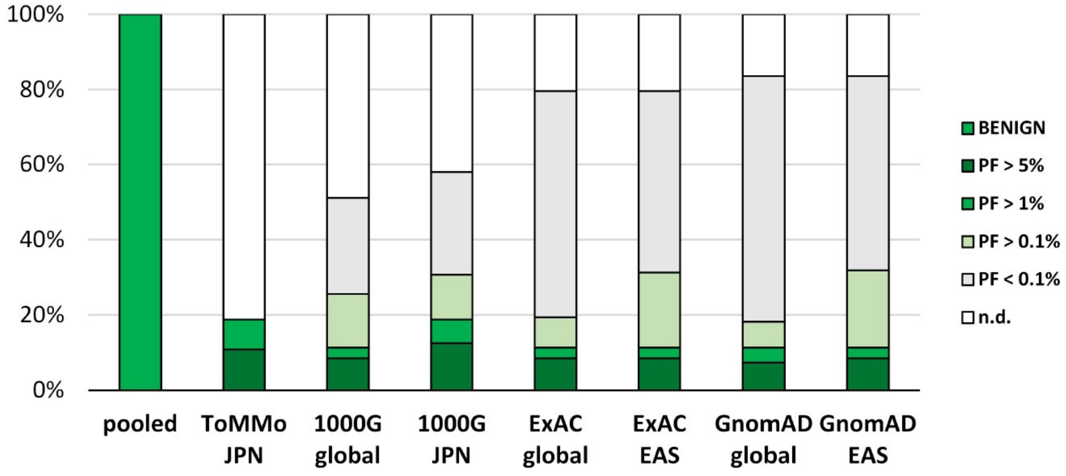

Figure S2. Comparison of Japanese-specific population-frequency data in ToMMo, 1000 Genomes, *ExAC*, and GnomAD. Benign classifications of 176 *BRCA1/2* variants were pooled together from four Japanese HBOC studies [12–15]. PF data of MH BRCA including the Japanese genomic cohort data of ToMMo were compared with the 1000 Genomes Project data (1000G), the Exome Aggregation Consortium (*ExAC*), and the Genome Aggregation Database (GnomAD). Abbreviation: PF = population-frequency; JPN = Japan; EAS = East Asia.

Supplemental Figure 3

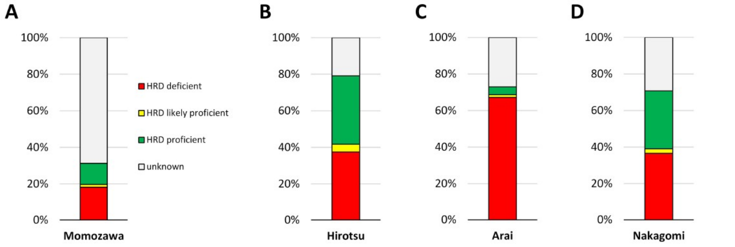

E

Total n= 1040	HRD deficient	HRD likely proficient	HRD proficient	n.d.
P/LP	P/LP	P/LP	P/LP	P/LP
282	269	11	1	1
B/LB	B/LB	B/LB	B/LB	B/LB
400	-	3	102	295
VUS	VUS	VUS	VUS	VUS
357	-	2	22	333

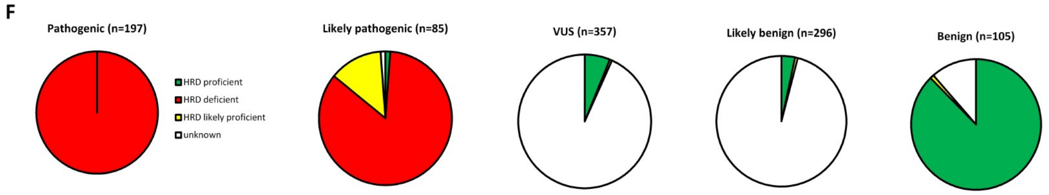

Figure S3. Correlation of MH BRCA classifications from Japanese HBOC studies with predicted response to PARP inhibition in MH Guide. (A–D) Predicted response to PARP inhibition in MH Guide of 1040 *BRCA1/2* variants collected from four Japanese HBOC studies [12–15] is based on the functional impact of neutral, hypermorphic, or hypomorphic variants in homologous recombination activity. (E,F) Cluster analysis of MH BRCA classified variants with predicted response to PARP inhibition in MH Guide within the sub-categories.
