# Supplementary material for: Consolidated BRCA1/2 Variant Interpretation by MH BRCA Correlates with Predicted PARP Inhibitor Efficacy Association by MH Guide
Source: Int J Mol Sci. 2020 May 29;21(11):3895. doi: 10.3390/ijms21113895 (PMC7312854; doi:10.3390/ijms21113895)
Supplement: Supplementary file 1 [file ijms-21-03895-s001.zip › Supplemental Table 3.pdf]

Table S3: Comparison of population-frequencies in ToMMo, 1000G, ExAC, or GnomAD

Selected examples of diversity in PF data between the investigated databases. Color code used for visualization: PF>5% (dark green), PF>1% (green), PF>0.1% (light green), and PF<0.1 % (white). Abbreviations: PF=population-frequency; JPN=Japan; EAS=East Asia

GENE	HGVS p.	SNP-ID number	ToMMo	1000G	1000G	ExAC	ExAC	GnomAD	GnomAD
			PF (JPN)	PF (Global)	PF (JPN)	PF (Global)	PF (EAS)	PF (Global)	PF (EAS)
BRCA2	S455=	rs1801439	12.63%	7.37%	8.65%	5.18%	10.03%	4.99%	10.12%
BRCA2	N289H	rs766173	12.63%	7.37%	8.65%	5.18%	10.02%	4.96%	10.19%
BRCA2	N991D	rs1799944	12.63%	8.00%	8.65%	5.34%	10.02%	5.22%	10.12%
BRCA2	H743=	rs1801499	12.63%	7.35%	8.65%	5.16%	10.02%	5.03%	10.13%
BRCA2	M784V	rs11571653	9.91%	0.36%	8.65%	0.03%	0.44%	0.02%	0.29%
BRCA1	K38=	rs1800062	7.88%	1.26%	10.10%	0.41%	4.14%	0.42%	3.91%
BRCA1	M1628T	rs4986854	1.90%	0.26%	5.29%	0.15%	0.64%	0.14%	0.42%
BRCA2	I3412V	rs1801426	1.89%	4.49%	0.96%	2.27%	2.60%	2.48%	2.43%
BRCA2	G2044V	rs56191579	1.44%	0.06%	1.44%	0.00%	0.06%	0.00%	0.05%
BRCA2	K2729N	rs80359065	1.39%	0.26%	1.44%	0.08%	0.97%	0.08%	0.92%
BRCA2	K322Q	rs11571640	1.35%	0.06%	1.44%	0.01%	0.08%	0.00%	0.05%
BRCA1	Y856H	rs80356892	-	0.30%	2.40%	0.15%	2.08%	0.16%	2.23%
